# Cognitive impairment in asymptomatic cerebral arterial stenosis: a P300 study

**DOI:** 10.1007/s10072-022-06442-9

**Published:** 2022-10-19

**Authors:** Nevine El Nahas, Amr Zaki, Magd Zakaria, Azza Abd El Naser, Ahmed El Bassiony, Eman Abdeldayem, Hossam Shokri, Ahmed El Bokl

**Affiliations:** Neurology Department, Faculty of Medicine, Ain Shams University, Cairo, Egypt

**Keywords:** Cerebral arterial stenosis, Asymptomatic cerebral arterial stenosis, Structural brain lesions, Cognitive dysfunction, Intracranial stenosis

## Abstract

**Background:**

Cerebral arterial stenosis (CAS), in the absence of a structural lesion, can result in cognitive impairment that represents an ongoing contention among studies. Accordingly, we investigated cognitive functions in asymptomatic patients with CAS, using P300 which is a neurophysiological tool. We also compared cognition in intracranial stenosis (ICS) and extracranial stenosis (ECS).

**Methods:**

Asymptomatic patients with CAS (≥ 70%) in the absence of structural brain lesions were categorized into ICS and ECS groups of 15 patients each, in addition to 15 normal controls. MRI, MRA, CT angiography, P300 analysis, Mini-Mental State examination (MMSE), Wisconsin Card Sorting Test (WCST), and Wechsler Memory Scale Test-Revised (WMST) were performed to all patients.

**Results:**

Impairment on all cognitive scales ranged from 70 up to 100% among CAS group. Prolonged p300 latency and reaction time correlated with worse performance on WMST (*p* = 0.02), while lower amplitude and decreased accuracy correlated with more errors on WCST (*p* = 0.01). ICS scores on WCTS were lower than those of ECS group (*p* = 0.001), while ECS had a longer reaction time (*p* = 0.02) and lower scores on MMS and WMST than those of ICS group (*p* = 0.03).

**Conclusion:**

Patients with asymptomatic CAS had a high prevalence of cognitive dysfunction which places them at risk of higher morbidity. ICS group showed impairment on executive functions, while the ECS group showed predilection to memory and information processing dysfunction.

## Introduction

Cerebral arterial stenosis (CAS) is known to impact the brain through various mechanisms, whether thromboembolic, luminal narrowing with impaired perfusion, or watershed infarction [1]. This might result in strokes that can either be overt and cause disability or silent so as to elude medical attention [2], 3. Moreover, the impact of CAS on the brain can even be more subtle and result in variable degrees of cognitive impairment, the consequences of which, as well as their management, are still controversial.

Zhao et al. [4] showed that the incidence of cognitive impairment in patients with moderate-to-severe stenosis was markedly higher than controls and could reach 79.7%.

Several other studies have investigated the influence of CAS on cognitive functions in patients with a history of overt as well as in silent cerebral infarctions where, in addition to stroke, the patients were found to suffer from impaired executive function and processing speed [2, 3, 5].

However, in patients with previous strokes, global ischemia due to CAS might not be the main player in cognitive decline since one cannot exclude the direct effect of a strategic infarction on cognition [5].

On the other hand, a systematic review revealed an ongoing debate about the prevalence of cognitive decline in cases of asymptomatic carotid stenosis [6].

Therefore, it seems that a contention still holds concerning the frequency and severity of cognitive decline as a consequence of CAS in the absence of a structural lesion. While some argue whether the benefit of revascularization outweighs its risks [7, 8], others claim that subclinical cognitive dysfunction in patients with CAS places them at risk of mobility problems leading to a *2.86 times greater* liability to falls [9]. Thus, early identification of covert cognitive impairment is essential to reduce the risk of institutionalization, morbidity, and mortality in elderly people with asymptomatic CAS. Also, in order to justify any form of intervention for CAS in asymptomatic subjects, we need to document cognitive impairment prior to intervention and furthermore to prove reversibility of impairment following revascularization.

Consequently, in the current study, we recruited patients having severe CAS with no structural brain lesions. All cases were either asymptomatic or had a TIA. Mental functions were assessed by psychometric tests. And since psychometric tests have shown disparity among studies, we also applied a visual odd ball test which is believed to have less practice effect and negligible test–retest variability than traditional scales [10].

Taghavy and Kiigler [11] (need PDF) showed that P300 is related to task processing and is thus an endogenous potential that is more biological and reproducible, with nearly no test–retest variability. We also investigated if intracranial arterial stenosis (ICAS) and extracranial arterial stenosis (ECAS) had a differential effect on cognitive functions.

## Methods

This is a cross-sectional case-controlled study where 30 consecutive participants were included. All patients had no history of cognitive impairment or enduring neurological deficit. Twenty-four cases presented with cardiac or peripheral vascular disorders and were referred for carotid duplex, and 6 patients gave a history of TIA. All of the patients had a normal MRI brain. All subjects gave an informed consent for participation, and the approval of the local IRB was obtained.

### Patients

The total number of the study population was 45, 15 with intracranial stenosis and 15 with extracranial stenosis and 15 age-matched healthy controls.

Patients were included if they were neurologically asymptomatic or had a history of TIA dating at least 3 months prior to recruitment. All patients had either intra- or extra-cranial severe arterial stenosis (≥ 70%) as diagnosed by MRA and confirmed by CT angiography, and all the patients and controls received at least 12 years of formal education. They were excluded if they or their relatives reported symptoms suggestive of cognitive impairment, concomitant neurological or metabolic disorders that could compromise mental functions, or if they had a history of a symptomatic infarction. Also, patients were excluded if MRI showed silent infarctions or small vessel disease.

### Procedures

1. Neurological history and examination were performed to verify inclusion and exclusion criteria. Laboratory investigations were done for vascular risk factors and echocardiography to exclude patients with low ejection fraction ≤ 50%.
2. Neuroimaging included magnetic resonance imaging (MRI) the brain including FLAIR sequence to ensure the absence of major infarctions and 3D TOF magnetic resonance angiography (MRA) to screen for the presence of intracranial stenosis. Carotid duplex of both carotids and vertebrobasilar system to detect extracranial stenosis.
  Patients showing arterial stenosis performed CT angiography for both extracranial and intracranial carotid and vertebrobasilar systems to confirm the presence and the degree of stenosis. CTA was conducted using a 64-slice CT scanner (Light Speed VCT 64- slice Scanner; General Electric, Milwaukee, WI). The degree of stenosis was calculated using the method published for the Warfarin-Aspirin Symptomatic Intracranial Disease Study: percent stenosis = [(1 − (*D* stenosis/*D* normal)] × 100, where *D* stenosis = the diameter of the artery at the site of the most severe stenosis and *D* normal = the diameter of the proximal normal artery [12].
3. Neuropsychological assessment included Mini-Mental State examination (MMSE), Wisconsin Card Sorting Test (WCST): to assess prefrontal lobe dysfunction [13], Wechsler Memory Scale Test-Revised (WMST) to assess different memory functions [14].

### P300 recording and analysis

Advanced Neuro’s EEGO sports amplifier and caps were used for EEG recording. Advanced Neuro-Technology e-evoked stimulus presentation software was used for stimulus presentation. Matlab — EEGlab/ERP lab (“ERPLAB Toolbox — ERPinfo.ORG,” n.d.) toolbox was used for ERP analysis [15].

EEG recording was performed using a 64-electrode cap with linked mastoid electrodes as a reference. The low-pass filter was 70 Hz, and the high-pass filter was 0.01 Hz with a sample rate 500 Hz, and impedance values were kept below 10 kΩ.

Visual oddball paradigm consisted of 200 trials where either of the two images was randomly presented (a man and a shark); one of the images was the frequent stimulus representing 85% of stimuli while the other was the rare (odd) stimulus representing 15% of stimuli. Each image was displayed on screen for 250 ms with an inter-stimulus interval randomly ranging from 1000 to 1100 ms.

Subjects were instructed to maintain fixation on the screen during the presentation of the stream and to press a button in a hand-held controller whenever they saw the rare stimulus. The timing of each stimulus and button presses was automatically marked and coded on the EEG by the stimulus presenting software.

#### P300 analysis

The EEG was segmented into epochs of 1200 ms with 200-ms pre-stimulus interval and 1000-ms post-stimulus interval. Baseline correction was applied to the pre-stimulus interval. Moving window technique was used for artifact rejection with a window size of 200 ms and a rejection threshold of 100 µv in all electrodes.

Independent component analysis (ICA) was done in all cases. Individual components were examined to remove eye, muscle, and movement artifacts. This was followed by averaging of all stimuli, and the waves for frequent and rare trials were subtracted, and the resultant wave was studied (frequent wave − rare wave = ERP wave).

P300 wave was identified as the largest positive peak occurring after the N1, P2, and N2 components in frontal to parietal scalp areas. In recordings with a bifurcated peak of Pa and Pb, Pb was considered to represent the P300 wave [16].

#### Definitions of wave components

*Amplitude* (μV) is defined as the difference between the mean pre-stimulus baseline voltage and the largest positive-going peak of the ERP waveform within a time window of 200–600 ms. *Latency* (ms) is defined as the time from stimulus onset to the point of maximum positive amplitude within the time window. *Reaction time* is calculated since the appearance of the odd-stimulus till controller button press within range of 200–1000 ms. *Accuracy* is defined as the percent of correct responses to the total number of odd stimuli. These wave components were automatically identified through ERP LAb toolbox to avoid human bias [17].

Patients’ results on cognitive scales were compared to normative data of normal elderly controls [13, 18–20]. And results of P300 odd ball test were compared to age-matched controls performed in the study.

### Statistical analysis

Statistical analysis was done using SPSS version 19th version Statistics (SPSS Inc., Chicago). To test for normality of continuous data distribution, the Shapiro-Wilks test was used. Mean and standard deviation were used for normally distributed data, while median and interquartile range (IQR) were used for skewed data. Categorical data were presented as frequencies. An independent samples *t*-test and one-way ANOVA (with Bonferroni post hoc test) were used to compare normally distributed continuous variable with nominal independent variables. The chi-square test was used for comparison of nominal data. The Pearson correlation coefficient was used to determine the relationship between two quantitative variables and the degree to which the two variables coincide with one another. For comparing patients with normative values for cognitive scales: the scores lying outside normal mean ± 2SD were considered abnormal.

## Results

A total of thirty patients were recruited, the mean age was 58 years, and males constituted 36.7%. The commonest risk factors were hypertension and diabetes, followed by dyslipidemia, smoking, and ischemic heart disease as shown in (Table 1).

**Table 1:** Descriptive results and clinical characteristics of the patient group

Variables	Patients (*N* = 30)		Controls (*N* = 15)	*p* value
	ICS (*N* = 15)	ECS (*N* = 15)		
Age, median (IQR)	62 (55–72)	55 (42–65)	63 (62–65)	0.09
Male, *n*. (%)	33.3%	40%	46.7%	0.8
Hypertension, *n*. (%)	73.3%	73.3%	80%	0.9
Diabetes, *n*. (%)	73.3%	73.3%	66.7%	0.9
Dyslipidemia, *n*. (%)	46.7%	13.3%	26.7%	0.1
Smoking, *n*. (%)	13.3%	26.7%	33.3%	0.4
	Patients (*N* = 30**)**		Normative values Mean (SD)	% patients deviated from norm
MMSE	24 (3.3)		27	70
WCST				
Total error	46.8 (12.4)		25.62 ± 20.87	83.3
Perseverative error	51.6 (12.7)		14.05 ± 13.43	100
WMST				
Total score	39.4 (8.7)		63 ± 12.6	100
Information	4.5 (1.4)		13.4 ± 0.6	100
Orientation	4.7 (1.3)			
Mental control	1.9 (1.6)		4.9 ± 1.2	93.3
Logical memory	7 (2.8)		22.5 ± 6.3	100
Digit span total	7.6 (2)		14.9 ± 3.3	90
Associate learning	3.9 (1)		6.9 ± 1.2	100
P300 latency (ms)	458.5 (89.6)		324 (87.1)	80
P300 amplitude (µv)	8.38 (4)		9.8 (2.8)	36.6
P300 reaction time (ms)	517.8 (50.3)		447.4 (26.4)	69.5
P300 accuracy (%)	86.9 (9.9)		95.5 (6.8)	47.8

*ICS* intracranial stenosis, *ECS* extracranial stenosis, *IQR* interquartile range, *MMSE* mini mental state examination, *WCST* Wisconsin card sorting test, *WMST* Wechsler memory scale test

The mean percentage of arterial stenosis was 88.3% with a predominance of anterior circulation stenosis representing 90% of the whole group.

The patients showed variable degrees of impairment on different cognitive scales and subscales ranging from 70 up to 100% (Table 1).

Eighty percent of patients showed delayed peak latency, and 69.5% showed delayed reaction time of P300, while P300 amplitude and accuracy were relatively less impaired (36.6% and 47.8%; respectively).

Comparison of the whole CAS group with controls and further comparison of ICS with ECS group showed non-significant difference as regards age, gender, degree of stenosis, and risk factors except for dyslipidemia that was more frequent in the ECS group; *p*: 0.04 (Table 2).

**Table 2:** Comparison of demographic data and risk factors of both test groups

	Groups		*p* value
	ICS *N* = 15	ECS *N* = 15	
Gender (male), %	6 (40%)	5 (50%)	1
Age (years), mean ± SD	54.06 ± 10.32	61.93 ± 10.78	0.05
Smoking, %	4 (26.7%)	2 (13.3%)	0.361
DM, %	11 (73.3%)	11 (73.3%)	1
HTN, %	11 (73.3%)	11 (73.3%)	1
ISHD, %	1 (6.7%)	5 (33.3%)	0.068
Dyslipidemia, %	2 (13.3%)	7 (46.7%)	0.04
Degree of stenosis, mean ± SD	87.2 ± 6.2	89.3 ± 5.9	0.359

*ICS* Intracranial stenosis, *ECS*, extracranial stenosis, *DM* diabetes mellitus, *HTN* hypertension, *ISHD* ischemic heart disease

### Assessment of cognitive functions in both test groups

The results of all cognitive scales were lower than the cut off values for normal elderly population. It is notable that the ICS group showed relatively worse scores than the ECS group on WCST that was only significant for total errors and perseverative errors (*p* ≤ 0.001 for each). The group of ECS displayed more decline on MMS, and on all categories of WMST, with a significant difference in mental control and total digital span scores (*p* = 0.03 for each) (Table 3).

**Table 3:** Assessment of cognitive functions in both groups

Cognitive Scales	ICS (*N* = 15)	ECS (*N* = 15)	*p* value	Normal cut off values
	Mean ± SD	Mean ± SD		Mean ± SD
MMSE	24.9 ± 3.7	23.1 ± 2.6	0.14	27
WCST				
Categories completed	2.2 ± 1.7	2.6 ± 1.6	0.52	5.07 ± 1.63
Total error	54.6 ± 8	38.4 ± 10.9	< 0.001	25.62 ± 20.87
Perseverative errors	53.2 ± 13.6	49.9 ± 11.9	< 0.001	14.05 ± 13.43
Non-perseverative errors	11.6 ± 6.6	13.1 ± 3.1	0.4	11.57 ± 9.79
WMST				
Total score	32.1 ± 9.3	27.7 ± 4.3	0.1	63 ± 12.6
Information	4.8 ± 1.5	4.3 ± 1.3	0.32	13.4 ± 0.6
Orientation	5.0 ± 1. 2	4.6 ± 1.2	0.38	
Mental control	2.5 ± 1.7	1.3 ± 1.4	0.03	4.9 ± 1.2
Logical memory	7.3 ± 3.2	6.6 ± 2.4	0.51	22.5 ± 6.3
Digit Span total score	8.4 ± 2.5	6.8 ± 1.0	0.037	14.9 ± 3.3
Associate learning	4.0 ± 1.3	3.8 ± 0.4	0.65	6.9 ± 1.2

*ICS* intracranial stenosis, *ECS* extracranial stenosis, *MMSE* mini mental state examination, *WCST* Wisconsin card sorting test, *WMST* Wechsler memory scale test

#### P300 analysis

**Fig. 1:**
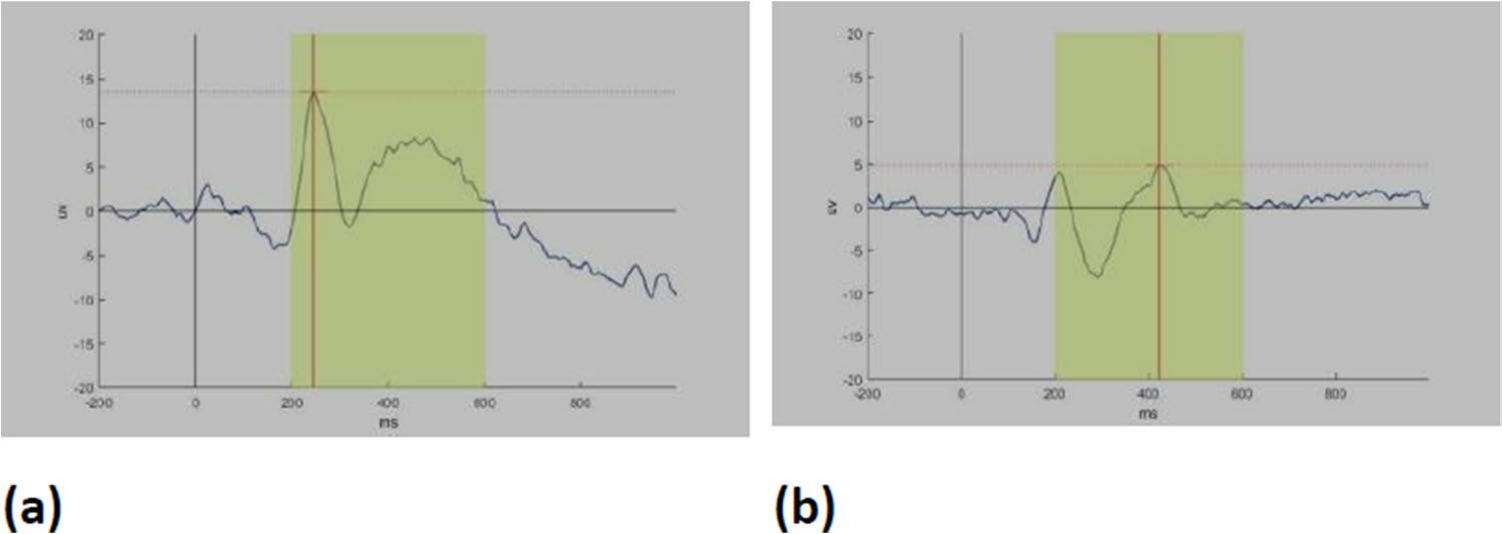
**a** P300 wave in a normal participant. **b** P300 wave in a patient with CAS showing delayed latency and diminished amplitude

Comparison of test groups with control group showed significantly prolonged latency, lower accuracy and longer reaction time (*p* ≤ 0.001, 0.006, and < 0.001; respectively) (Fig. 1). Post hoc analysis to compare each test group to normal showed that ECS group had a significantly longer latency, less percentage of accuracy, and longer reaction time (*p* ≤ 0.001, 0.005, and < 0.001 respectively). The ICS group showed significantly longer latency and reaction time than the control (*p* = 0.004 and 0.005 respectively).

Comparison of both test groups showed that ECS group had a significantly longer reaction time (*p* = 0.02), with a relatively longer latency and lower accuracy than ICS group (Table 4).

**Table 4:** Analysis of P300 in both test groups and control group

	Intracranial stenosis *N* = 15		Extracranial stenosis *N* = 15		Control group *N* = 15		*p* value
	Mean	SD	Mean	SD	Mean	SD	
Latency (ms)	434.8	98.0	482.2	79.8	324.0	87.1	< 0.001
Amplitude (µv)	7.4	2.9	12.1	11.0	9.8	2.8	0.182
Accuracy (%)	90.0	4.9	85.2	11.1	95.5	6.8	0.006
Reaction time (ms)	493.1	39.6	530.9	43.4	447.4	26.4	< 0.001
Post hoc Bonferroni		ICS vs ECS *p* values			ICS vs control *p* values		ECS vs control *p* values
Latency (ms)		0.45			0.004		< 0.001
Accuracy (%)		0.35			0.22		0.005
Reaction time(ms)		0.02			0.005		< 0.001

*ICS* intracranial stenosis, *ECS* extracranial stenosis

Correlation between P300 parameters and neuropsychological scales showed that P300 latency negatively correlated with total Wechsler Memory Score, logical memory, and digital span: *p* = 0.02, 0.02 and 0.04; respectively. P300 amplitude negatively correlated with perseverative errors: *p* = 0.01. P300 reaction time negatively correlated with total Wechsler memory score, mental control, and digital span: *p* = 0.007, 0.002, and 0.02. P300 accuracy negatively correlated with non-perseverative errors (Table 5).

**Table 5:** Correlation between P300 parameters and neuropsychological scales (WMST, WCST)

Cognitive scales		P300 Latency	P300 amplitude	P300 reaction time	P300 accuracy
MMSE	Pearson	− .299	− .279	− .238	− .216
	Sig	0.1	0.13	0.2	.252
Categories completed	Pearson	− .117	.155	− .098	− .258
	Sig	0.53	0.4	0.6	.169
Perseverative errors	Pearson	− .128	− .455	− .002	− .014
	Sig	0.5	0.01	0.9	.941
Non-perseverative errors	Pearson	− .188	.026	.311	− .410
	Sig	0.32	0.89	0.09	0.025
Total score (WMST)	Pearson	− .414	− .155	− .486	.071
	Sig	0.02	0.4	0.007	0.7
Information	Pearson	− .283	.020	− .272	− .139
	Sig	0.12	0.9	0.14	0.46
Orientation	Pearson	− .248	.220	− .302	.112
	Sig	0.18	0.24	0.1	0.55
Mental control	Pearson	− .250	− .324	− .536	.079
	Sig	0.18	0.08	0.002	0.67
Logical memory	Pearson	− .415	− .200	− .361	.067
	Sig	0.02	0.28	0.05	0.72
Digit Span total score	Pearson	− .369	− .107	− .424	.106
	Sig	0.045	0.57	0.02	0.57
Associate learning	Pearson	− .098	− .200	− .097	.004
	Sig	0.6	0.29	0.6	0.98

*MMSE* mini mental state examination, *WCST* Wisconsin card sorting test, WMST Wechsler memory scale test

To sum up:

- *Prolonged latency and prolonged reaction time correlated with worse performance on WMST.*
- *Lower amplitude and decreased accuracy correlated with more errors on WCST.*

## Discussion

Previous research has shown discordant results concerning the impact of CAS on cognition. While various studies showed that CAS was significantly associated with bad performance on executive functions and memory [21–23], others found no such association [24, 25].

In the current study, all the patients had severe stenosis, and their cognitive scores were markedly inferior to normative scores, despite that none of them complained of cognitive impairment. Similar findings were described by Jackson and colleagues [26] and others [22]. Liu et al. reported that up to 79.7% of his cohort of ICS had cognitive dysfunction [27].

The controversial results for cognitive affection with CAS could be explained in the context of variation in cerebral vasomotor reactivity among patients with asymptomatic stenosis. Patients with impaired reactivity were more liable to have cognitive impairment [28]. Moreover, those with reduced perfusion had a worse performance on MMSE test. However, others attributed cognitive deterioration in patients with CAS to non-disabling strokes [5].

In the current study, the impact of stroke or any structural lesion on cognition has been precluded by history and by MRI. Thus, any degree of impairment could be ascribed to the global effect of CAS.

All the patients showed variable degrees of impairment of executive as well as memory functions. We should however be conservative while interpreting low scores on memory tests that are observed in the presence of impaired mental control. Mental control measures attentional abilities; thus, the defective memory performance could be ascribed to inadequate information acquisition [14].

This uncertainty could be verified by the P300 study which is a more objective electrophysiological test, less dependent on patients’ motivation, and less liable to learning effect. P300 reflects information processing which, according to the information processing theory, is an integral part of memory function, whether sensory, working, or long-term memory [29]. Similarly, other researchers employed different neurophysiological tools as transcranial magnetic stimulation to study the therapeutic effect of pharmacological intervention on cognitive functions in patients with mild cognitive impairment [30].

We included an age-matched normal control group to avoid the reported variability of P300 parameters according to age and literacy, in addition to the paucity of research that targets healthy elderly [31]. Compared to normal controls, patients with CAS showed prolonged P300 latency and reaction time which reflect delayed information processing, while accuracy and amplitude, which refer to restricted attention and concentration, were less impaired. This discrepancy might denote actual impairment of information processing rather than attention. Similar findings were reported where clinically non-demented patients with high-grade stenosis had longer latency and lower amplitude [32] that correlated with cognitive testing [10].

Delayed P300 latency and prolonged reaction time, in our patients, were correlated with bad performance on WMST. P300 latency is known to represent information processing, and delayed elements of this endogenous wave are related to memory dysfunction [10].

On the other hand, reduced amplitude and accuracy were correlated with WCST. Amplitude and accuracy reflect impaired attention which is an integral part of executive functions [33]. These findings were similarly reported showing that different parameters of P300 wave coincided with cognitive testing [33, 34].

Consequently, we can assume that in our patients, both processes of mental control and memory function are defective.

When the patients were compared according to the site of stenosis, the ICS group had more impairment on executive functions, while the ECS group showed more memory dysfunction. Also, while both groups were defective on visual oddball testing, ECS had a significantly worse performance. It has been previously demonstrated that severe ECS can compromise the development of collaterals that are required to compensate for hypoperfusion [8] and might result in a state of global dys-autoregulation [35].

On the other hand, ICS being more distal, allows for such collaterals to develop from the patent proximal sites [36]. Thus, ECS is expected to result in more cognitive dysfunction than ICS.

Finally, we would like to highlight that in order to explore the reversibility of cognitive dysfunction in this group of patients, they receive revascularization by angioplasty. The impact of this intervention on cognitive functions will be reported in another article.

## Conclusion

Patients with asymptomatic CAS have a high prevalence of cognitive dysfunction which places them at risk of higher morbidity. ICS group had more impairment on executive functions, while the ECS group showed more memory and information processing dysfunction. Early detection of stenosis with significant effect on cognition, even before the incidence of stroke, would maximize the clinical benefit of vascular interventions in these patients.

### Study limitations

Among the limitations of this research is the lack of perfusion studies for cases. This would have added valuable information about the state of global brain perfusion in asymptomatic patients and its correlation with cognitive impairment.
